# The Treatment of Toxemia of Pregnancy as Based on the Study of Seventy-eight Cases

**Published:** 1907-06

**Authors:** 


### THE TREATMENT OF TOXEMIA OF PREGNANCY AS BASED ON THE STUDY OF 78 CASES.

W. H. Wells says: The treatment of the taxemia of pregnancy is based on efforts to increase elimination, first of all by the bowels. Purgation should be started and continued until the bowels are absolutely clean and a good flow of that natural intestinal antiseptic, the bile, is started. For this purpose calomel given in doses of 1-4 grain every hour until 10 grains are taken, and often combined with one-fourth to one-half a drop of croton oil, and followed by the free use of salines, gives in our experience the best results. If there is vomiting, or to relieve the substernal distress so often complained of, lavage of the stomach may be practiced with good effect. The stomach may be washed out with sterile water with bicarbonate of soda 3 drahms to the quart. If the calomel produces nausea when given by the mouth, it may be washed into ehe stomach through the tube. Unload the lower bowel by washing out the colon, first by the usual "triplex enema," then by lavage of normal salt solution. The lavage should be repeated three or four times a day until the solution comes out clean. After the bowels have been thoroughly emptied the enteroclysis should be discontinued. I have frequently found it a very difficult matter to thoroughly empty the intestine of a pregnant woman. Following the enteroclysis the intestinal peristalsis should be stimulated by such drugs as nux vomica, cascara, and possibly aloes, while a mild impression by calomel may be kept up for some time after the attack is over and strict attention should be given to the bowes until the end of pregnancy.

Excretion through the skin should be stimulated in mild cases of toxemia by simple warm baths, proper clothing, and exercise. In cases more severe the hot pack will be necessary. These packs must be continued until a free perspiration is produced. To quiet the high-tensioned circulation, bromides or chloral in suitable doses may be used, or the tincture of veratrum viride in doses of 15 to 20 minims is better. When the nervous tension is high a hypodermic injection of 1-4 grain of morphine does good and may be repeated once. It quiets the patient's restlessness until the eliminatives have time to work.

To increase the flow of urine and favor excretion by the kidneys, the patient should drink a reasonable quantity of water, say two quarts a day (in twenty-four hours); the liquid foods also favor diuresis. Sweet spirits of nitre, citrate of soda or lithium, acetate of potash, salicylate of soda, are all of use.

The diet should be restricted to milk and mild foods, the lighter broths, and fruit during the attack. Later the white meat of chicken may be given. All stimulants should be prohibited, as they do nothing but harm. Even tea and coffee are better done without.

Plenty of fresh air is a necessity to these patients—at first in the patient's room; and later a regulated amount of exercise should be taken daily. Massage and passive movements are of use. We have never used hypodermoclysis or bloodletting unless eclampsia develops, and then there is no doubt of their usefulness in suitable cases.

As a rule we do not believe the induction of permature labor helps in the cure of toxemia where the latter comes on late in pregnancy. Certainly at least it should not be considered unless all other means of cure fail. If labor comes on spontaneously I do nothing to prevent it, but if the patient does not develop eclampsia spontaneous premature labor in my experience has been somewhat rare. The forced induction of labor seems to me to simply add the shock of a rather severe obstetric operation, and should not be considered except as a last resort. In case of toxemia arising early in pregnancy, especially when the disease manifests itself in pernicious nausea, it may be necessary to empty the uterus promptly.

In a large number of cases the toxemia will respond to proper treatment, so that interruption of the pregnancy will be unnecessary. In the toxemia of pregnancy as in many other diseases the value of the treatment depends a great deal on the earliness of its instigation; the earlier the treatment is started the better the prognosis.—Therapeutic Gazette, April 17.

